# Phosphodiesterase-4 Inhibition in the Management of Psoriasis

**DOI:** 10.3390/pharmaceutics16010023

**Published:** 2023-12-22

**Authors:** Erika L. Crowley, Melinda J. Gooderham

**Affiliations:** 1 Faculty of Medicine, University of British Columbia Okanagan, 3333 University Way, Kelowna, BC V1V 1V7, Canada; erikalc@student.ubc.ca; 2 SKiN Centre for Dermatology, 775 Monaghan Rd, Peterborough, ON K9J 5K2, Canada; 3 Probity Medical Research, 139 Union St E, Waterloo, ON N2J 1C4, Canada; 4 Department of Medicine, Queen’s University, 99 University Ave, Kingston, ON K7L 3N6, Canada

**Keywords:** psoriasis, PDE4 inhibitor, apremilast, roflumilast, crisaborole, clinical trials, review

## Abstract

Psoriasis is a common chronic immune-mediated disease with many comorbidities and impacts on quality of life. Among the treatments for psoriasis, phosphodiesterase-4 (PDE4) inhibitors are emerging with expanding options. PDE4 inhibitors play a pivotal role in the inflammatory cascade by degrading cyclic adenosine monophosphate (cAMP), contributing to pro-inflammatory mediator production. Apremilast, an oral PDE4 inhibitor, is approved for psoriasis. While effective, its adverse effects can limit its utility. Roflumilast, a topical PDE4 inhibitor, was also recently approved for psoriasis and shows promise in clinical trials. Crisaborole, a PDE4 inhibitor approved for atopic dermatitis, has also been studied in psoriasis. This review summarizes evidence from randomized clinical trials regarding the efficacy and safety of PDE4 inhibitors in psoriasis treatment. By highlighting their potential benefits and limitations, this review provides valuable insights for clinicians and researchers aiming to optimize psoriasis management.

## 1. Introduction

Psoriasis, a chronic immune-mediated inflammatory skin disorder, has many subtypes. Plaque psoriasis (or psoriasis vulgaris) is the most prevalent, affecting approximately 90% of psoriatic patients [1]. It is characterized as well-demarcated, erythematous to violaceous plaques depending on the skin tone, covered with scales. Psoriasis typically involves extensor surfaces but can also involve other parts of the body including the scalp, face, and intertriginous regions [2]. Pruritus is the symptom most bothersome in nearly half of patients [3]. Psoriasis affects individuals of all ages with a global prevalence of 0.5 to 11.4% in adults and 0 to 1.4% in children [4]. The condition significantly impacts Quality of Life (QOL), contributing to elevated rates of self-esteem issues, sexual dysfunction, anxiety, depression, and suicidal ideation [5,6]. Impact on QOL is greater with special site involvement such as face, groin, palms, and soles [3]. Additionally, there are associated comorbidities, including psoriatic arthritis, obesity, metabolic syndrome, and atherosclerotic disease [7].

Current treatment options for psoriasis encompass topical corticosteroids, retinoids, calcipotriol, and oral and injectable systemic therapies [8]. These treatment modalities range from topicals to broad immunosuppressive systemic agents and targeted biological therapies that inhibit specific cytokines or receptors, thereby controlling downstream inflammation. Treatment decisions are typically guided by disease severity, with mild cases managed using over-the-counter skin care products and topical therapies and moderate-to-severe cases necessitating systemic interventions. Topical treatments, in particular, can be challenging from an adherence perspective [9]. Given the substantial burden of psoriasis, there is a pressing need for innovative treatment options. One expanding avenue of interest is the inhibition of phosphodiesterase 4 (PDE4). Previous reviews have examined the emerging role of PDE4 inhibition in psoriasis including clinical trial data [10]. In this review, we aim to provide a comprehensive updated overview of psoriasis pathophysiology, the role of PDE4 inhibition, and the efficacy and safety of PDE4 inhibitors with a focus on the randomized clinical trials.

## 2. Psoriasis Pathogenesis

The pathogenesis of psoriasis is immune-mediated inflammation resulting from a complex interplay of immune dysregulation, genetic predisposition, and environmental factors [11]. This condition is characterized by the dysregulation or alteration of both innate and adaptive immune responses, as well as aberrant keratinocyte function and vascular changes [12]. The inflammatory cascade in psoriasis is driven by plasmacytoid dendritic cells (pDCs) and myeloid dendritic cells (mDCs), leading to the production of various inflammatory mediators, including tumour necrosis factor (TNF)-α, interleukins (IL)-12, IL-17, IL-22, IL-23, and interferon (IFN)-γ [13].

Plasmacytoid dendritic cells stimulate the adaptive immune response through the activation of T cells, primarily via IFN-α [12]. Activated macrophages produce IL-12 and IL-23, while myeloid dendritic cells contribute to the development of T helper type 17 and T helper type I cells, which, in turn, produce IL-17 and IL-22. These cytokines play a crucial role in promoting keratinocyte activation and growth. Importantly, while psoriatic plaques in the epidermal layer of the skin are the hallmark clinical manifestation of psoriasis, the inflammatory response involves intricate interactions between keratinocytes and various immune cell types within the dermal layer of the skin [12].

Cyclic adenosine monophosphate (cAMP), an essential secondary messenger ubiquitously found in all cells, assumes a critical role in a multitude of critical cellular processes [10,14]. The initiation of cAMP activation commences with G-protein coupled receptor ligands, which facilitate signal transduction predominantly through protein kinase A (PKA). Subsequently, PKA activates the cAMP response element-binding protein (CREB), exerting control over pivotal genes associated with psoriasis, such as IL-2, IL-6, IL-10, and TNFα [15].

## 3. Phosphodiesterase-4 and Inhibition

**Figure 1:**
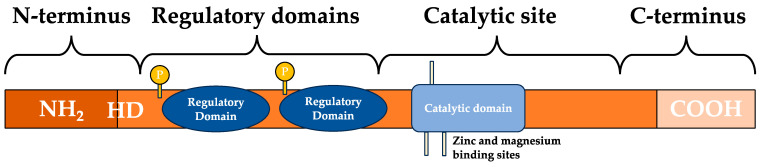
General structure of PDE enzymes, adapted from [17]. COOH—carboxyl group; C-terminus—carboxyl terminus; HD—hydrophobic domains; N-terminus—amino terminus; NH_2_—amino group; P—phosphorylation.

Phosphodiesterase (PDE) encompasses eleven enzyme families (PDE1-PDE11) responsible for terminating cyclic nucleotide signalling by catalyzing cAMP and cGMP hydrolysis [16,17]. PDE4, PDE7, and PDE8 specifically target cAMP, while PDE5, PDE6, and PDE9 focus on cGMP. PDE1, PDE2, PDE3, PDE10, and PDE11 exhibit dual specificity for both cAMP and cGMP, depending on the isoform hydrolysis [17,18]. As seen in Figure 1, these PDEs share common structural components, including a conserved catalytic domain, a regulatory domain between the amino (N) terminus and catalytic domain, and a region that can be phosphorylated (PDE4) or prenylated (PDE6) between the catalytic core and the carboxyl (C) terminus [17,19]. The N-terminal portion of PDE molecules determines their specific properties, including subcellular localization, interactions with signalosomes, and responses to regulatory signals [17].

PDE4, the largest and earliest discovered cAMP-specific PDE family, has four members including PDE4A, PDE4B, PDE4C, and PDE4D [18,20]. PDE4s are expressed in many different tissues to varying degrees including the brain, cardiovascular tissues, smooth muscles, keratinocytes, and immune cells like lymphocytes, granulocytes, and monocytes/macrophages [17]. When PDE4 catalyzes the hydrolysis of cAMP in these tissues, activation commences with G-protein coupled receptor ligands to facilitate signal transduction through PKA and activate CREB [20]. This exerts control over the genes that can be associated with psoriasis to start inflammatory cytokine secretion (e.g., IFN-γ, TNF-α, IL-2, IL-12, IL-23) [21].

Consequently, the inhibition of PDE4 stops the hydrolysis of cAMP, increases intracellular concentrations of cAMP, and therefore reduces the end product inflammatory cytokine secretion, suppresses superoxide generation, and inhibits chemotaxis [10]. PDE4s can also upregulate anti-inflammatory cytokines like IL-10 through CREB activation [15,22]. The concept of utilizing cAMP as a therapeutic approach for psoriasis continues to hold promise in the current medical landscape [10].

## 4. Phosphodiesterase-4 Inhibitors

PDE4 inhibitors are small molecules that can be selective or nonselective for cAMP [23]. Non-selective PDE4 inhibitors have no known benefit for psoriasis but selective PDE4 inhibitors have found application in the treatment of various dermatologic inflammatory conditions, including but not limited to psoriasis, cutaneous sarcoidosis, discoid lupus erythematosus, scleroderma, Behcet’s disease, and atopic dermatitis [10,14]. Over the years, a multitude of PDE4 inhibitors have been identified, with some of these compounds advancing to clinical trials [24,25]. Please refer to Table 1 and Table 2 for an overview of the clinical trials conducted in the context of oral and topical PDE4 inhibition for psoriasis with sponsors, clinical trial phases, and National Clinical Trial (NCT) numbers listed. We will emphasize the most extensively studied PDE4 inhibitors for psoriasis including apremilast, roflumilast, and crisaborole and then briefly discuss other PDE4 inhibitors that have been investigated.

### 4.1. Apremilast

Apremilast (CC-1004; Otezla^®^) is an oral PDE4 inhibitor (Table 1 and Table 3). It was the inaugural PDE4 inhibitor to receive Food and Drug Administration (FDA) approval for the treatment of moderate-to-severe plaque psoriasis, psoriatic arthritis, and Behcet’s disease in 2014 [37]. It has since received approval in multiple countries, including Canada, the United States, European Union, and Japan [45]. Clinical trials have contributed to an understanding of apremilast’s efficacy and safety in a variety of dermatologic conditions [25]. Clinical trials of apremilast for psoriasis have been completed in phases 2, 3, and 4, which are listed in Table 1.

**Table 2:** Topical phosphodiesterase-4 inhibition clinical trials completed for psoriasis. Information retrieved from [25].

PDE4 Inhibitor	Formulation	Sponsor	Condition	Phases	NCT Number (Year Completed; Bolded Randomized Control Trials)
**Roflumilast (ARQ-151)**	Topical	Arcutis Biotherapeutics, Inc. (Westlake Village, CA, USA)	Psoriasis, plaque psoriasis, scalp psoriasis	3	NCT05684744 (2023); **NCT05763082 (2023); NCT05028582 (2022) [46]; NCT04211389 (2020) [47]; NCT04211363 (2020) [47]**
				2	NCT05684744 (2023); NCT04746911 (2022); **NCT04549870 (2022) [48];** NCT04746911 (2022); NCT04655313 (2022); NCT03764475 (2020 [49]); NCT04128007 (2020); **NCT03638258 (2019) [50]; NCT03392168 (2018) [51]**
				1	NCT04279119 (2021); **NCT03392168 (2018) [51]**
**Crisaborole (AN2728)**	Topical	Pfizer (Albany, GA, USA)	Plaque-type psoriasis; Psoriasis	2	**NCT01300052 (2011); NCT01029405 (2010)** **NCT00759161 (2008 [52]); NCT00755196 (2008)**
				1	**NCT01258088 (2010); NCT00763204 (2008)** **NCT00762658 (2007)**
**PF-07038124**	Topical	Pfizer	Plaque Psoriasis	1	**NCT05375955 (2023); NCT04664153 (2021)**
**Leo-29102**	Topical	Leo Pharma (Moscow, Russia)	Psoriasis vulgaris	2	**NCT00875277 (2009)**
				1	NCT01466478 (2011)
**MK-0873**	Topical	Merck Sharp & Dohme LLC (Rahway, NJ, USA)	Psoriasis; plaque psoriasis	1	**NCT01140061 (2011); NCT01235728 (2011)**
**DRM02**	Topical	Dermira (Menlo Park, CA, USA)	Plaque psoriasis	2	**NCT01993433 (2014)**

NCT—National Clinical Trial.

Among the earliest trials, a 12-week phase 2 randomized, double-blind, placebo-controlled clinical trial (NCT00606450) reported by Papp et al. (2013) [41] demonstrated the efficacy of apremilast at a dosage of 20 mg BID in addressing moderate-to-severe plaque psoriasis. This trial, involving 259 subjects, led to a higher proportion of patients treated with apremilast achieving a 75% or greater reduction in the Psoriasis Area and Severity Index >75% (PASI-75) compared to those on placebo. Furthermore, the reported adverse events were predominantly of mild-to-moderate severity including headache, nasopharyngitis, diarrhea, and nausea, with no severe adverse outcomes documented. Another early investigation, a phase 2b dose-ranging study (NCT00773734) reported by Papp et al. (2012) [40], enrolled 357 patients. This placebo-controlled trial corroborated the effectiveness of apremilast, especially at doses of 20 or 30 mg administered twice daily. It revealed a substantial increase in the proportion of patients attaining a PASI-75 at the 16-week mark when compared to the placebo. Importantly, the associated adverse events remained predominantly mild or moderate, including nausea, upper respiratory tract infection, diarrhea, nasopharyngitis, headache, gastroenteritis, or dyspepsia with no adverse impact on various clinical parameters [40]. These studies [40,41] laid the groundwork for phase 3 trials.

Two pivotal phase 3 multicentered, randomized, double-blinded, placebo-controlled trials, designated ESTEEM 1 (NCT01194219 [38]) and ESTEEM 2 (NCT01232283; [37]), represent milestones in the understanding of apremilast for moderate-to-severe plaque psoriasis. Subjects had psoriasis, PASI ≥ 12, body surface area (BSA) ≥ 10%, and physician global assessment (PGA) ≥ 3. According to Papp et al. (2015) [38], ESTEEM 1 enrolled 844 subjects and the administration of apremilast at 30 mg twice daily was the primary intervention. A substantial proportion of patients achieved a PASI-75 response (33.1%), surpassing the placebo group (5.3%, *p* < 0.0001), at the 16-week juncture. In the following 16 weeks, patients who received the placebo were switched to apremilast 30 mg twice daily with subsequent improvement in their PASI-75 and pruritus visual analogue scale (VAS) at week 32. The durability of these therapeutic benefits was evident as most patients (61.0%) rerandomized to receive apremilast at week 32 maintained their PASI-75 response at week 52 compared to those rerandomized to the placebo (11.7%). The safety profile of apremilast continued to demonstrate a favourable trend, with adverse events predominantly being of mild-to-moderate intensity and without new significant adverse events. Following ESTEEM 1, ESTEEM 2 enrolled 411 patients and reinforced the efficacy of apremilast at 30 mg twice daily [37]. The trial not only witnessed a higher proportion of patients achieving PASI-75 (28.8%), but also Psoriasis Area and Severity Index ≥50% (PASI-50) (55.5%), static PGA score of 0 or 1 (20.4%), and Dermatology Life Quality Index (DLQI), all in comparison to those receiving the placebo at week 16 (5.8%, 19.7%, 4.4%, respectively, *p* < 0.001). Patients rerandomized to continue the apremilast treatment maintained their PASI-50 response at week 52 (80%). The incidence of adverse events exhibited no increase during the 52-week treatment period, remaining consistent with prior reports. In ESTEEM 2, there was weight loss > 5% in 20.2% of the patients and two patients discontinued treatment as a result.

In a secondary analysis of the ESTEEM trials, Rich et al. (2016) assessed the effectiveness of apremilast in treating challenging nail and scalp psoriasis in 1255 adults [58]. During the initial 16 weeks, patients receiving apremilast exhibited significant improvements in Nail Psoriasis Severity Index (NAPSI) scores compared to those on a placebo (ESTEEM 1: −22.5% vs. +6.5%, *p* < 0.0001; ESTEEM 2: −29.0% vs. −7.1%, *p* = 0.0052). Additionally, the Scalp Physician Global Assessment (ScPGA) response was significantly more favourable with the apremilast treatment (*p* < 0.0001). Notably, patients who initially responded to PASI and received apremilast or a placebo maintained a 50% improvement in NAPSI (NAPSI-50) during follow-up (ESTEEM 1: 70.7% and 64.1%, ESTEEM 2: 68.6% and 69.0%, respectively). This finding also applied to patients with scalp psoriasis.

Subsequent to the ESTEEM trials, Reich et al. (2017) reported the phase 3b, multicentred, double-blinded, randomized controlled study called LIBERATE (NCT01690299) to assess apremilast and etanercept in moderate-to-severe plaque psoriasis with 250 subjects [36]. At week 16, apremilast demonstrated a significant achievement of PASI-75 (39.8%) compared to a placebo (11.9%, *p* < 0.0001), and this clinical benefit was sustained over 104 weeks. LIBERATE also evaluated the safety and effectiveness of transitioning from etanercept to apremilast (PASI-75 maintained 45.9–51.9%). Adverse events related to apremilast were primarily of mild or moderate severity consistent with previous reports of weight loss (mean −0.78 kg apremilast; +0.03 kg placebo) and one patient with psychotic disorder and suicidal ideation. The study demonstrated that the transition from etanercept to apremilast was well-tolerated, with no new safety or tolerability issues observed throughout the evaluation period.

The UNVEIL study (NCT02425826) was reported by Strober et al. (2017) [30] and Stein Gold et al. (2018) [29]. UNVEIL was a phase 4 double-blinded, randomized, placebo-controlled trial with 136 participants with systemic-native chronic moderate plaque psoriasis (BSA 5–10%, sPGA 3) including lower psoriasis. Over the first 16 weeks, apremilast 30 mg twice daily exhibited significant improvements in 75% improvement in the product of PGA and BSA(PGAxBSA-75) (−48.1%) and DLQI (−4.8) in comparison to the placebo group (−10.2%, *p* < 0.0001; −2.4, *p* = 0.0008 respectively). Notably, patients reported higher treatment effectiveness and global satisfaction with apremilast. Adverse events remained primarily of mild or moderate severity, with common side effects consistent with previous trials.

In a phase 4 double-blinded, randomized, placebo-controlled study (NCT02400749) reported by Bissonnette et al. (2018), 100 patients with moderate-to-severe palmoplantar psoriasis were enrolled [28]. Although no significant difference in the proportion of patients achieving a Palmoplantar Psoriasis Physician Global Assessment (PPPGA) of 0/1 at week 16 was observed between the apremilast and placebo groups, several secondary endpoints favoured apremilast. These secondary endpoints included improvements in the Palmoplantar Psoriasis Area Severity Index (PPPASI) (22%), DLQI (−4.3 ± 5.1) and activity impairment (−11 ± 22.3) compared to the placebo (8%, *p* = 0.0499; −4.3 ± 5.1, *p* = 0.00004; 2.5 ± 25.5, *p* = 0.0063). Adverse events were consistent with previous reports.

Van Voorhees et al. (2020) reported a phase 3b double-blinded, randomized, placebo-controlled study, including 303 subjects (STYLE, NCT03123471) [35]. Apremilast was evaluated for the treatment of moderate-to-severe scalp psoriasis in adults who had not adequately responded to topical scalp psoriasis therapies. Apremilast demonstrated a significant proportion of patients achieving improved SPGA (43.3% vs. 13.7% with placebo, *p* < 0.001), reduced the whole body itch numeric rating scale (NRS) (45.5% vs. 22.5% with placebo, *p* < 0.001), reduced scalp NRS (47.1 % vs. 22.5% with placebo, *p* < 0.001), and improved DLQI (−6.7 vs. −3.8 with placebo, *p* < 0.0001). Common adverse events were consistent with previous reports including depression and mean weight loss −0.9 kg vs. −0.2 kg in placebo.

Stein Gold et al. (2022) reported a double-blinded, randomized, placebo-controlled phase 3 study (ADVANCE; NCT03721172) featuring 595 subjects [33]. Apremilast was administered at a dose of 30 mg twice daily for patients with mild-to-moderate psoriasis who had exhibited an inadequate response or intolerance to topical psoriasis therapies. Apremilast demonstrated efficacy, as evidenced by a significantly higher proportion of patients achieving greater sPGA (21.6% vs. 4.1%, *p* < 0.0001), BSA-75 (33.0% vs. 7.4%), BSA <3% (61.0% vs. 22.9%), >4-point reduction in whole body itch NRS (43.2% vs. 18.6%), and scalp PGA (44.0% vs. 16.6%). Additionally, improvements were observed across QOL. Common adverse events associated with apremilast aligned with prior studies.

Recently, the EMBRACE study (NCT03774875), reported by Mrowietz et al. (2023), was a phase 4, multicenter, randomized, placebo-controlled study of 277 patients. The impact of apremilast, administered at a dose of 30 mg BID, on QOL, efficacy, and safety in patients with plaque psoriasis in visible locations, scalp, nails, genitals, and palmoplantar regions was assessed. The study encompassed patients with limited skin involvement who had not responded to topical therapy or conventional systemic treatment. Apremilast emerged as a significant enhancer of skin-related QOL (73.3% vs. 41.3% placebo, *p* < 0.0001). It also exhibited greater efficacy than the placebo in affected BSA (−19.8% vs. 18.5 placebo, *p* = 0.0085), PASI < 3 (39.7% vs. 26.3% placebo, *p* = 0.0328), itch NRS (−2.5 vs. −0.9 placebo, *p* < 0.0001), and skin discomfort/pain VAS (−21.5 vs. −5.4, *p* = 0.0003) at week 16. The safety profile of apremilast was consistent with previous studies: mild-to-moderate with no treatment discontinuation.

Results from the DISCREET study (NCT03777436) were presented at the 31st Annual Meeting of the European Academy of Dermatology and Venereology by Merola et al. (2022) [32]. This phase 3 randomized, placebo-controlled trial of 289 patients was designed to evaluate the impact of apremilast on patients dealing with moderate-to-severe genital psoriasis over a 16-week period. Apremilast demonstrated a significantly superior response in comparison to a placebo regarding genital psoriasis sPGA (39.6% vs. 19.5% placebo), genital itch, and DLQI. Adverse events were consistent with previous reports.

Lastly, results from the SPROUT study (NCT03701763) were also presented at the 31st Annual Meeting of the European Academy of Dermatology and Venereology by Fiorillo et al. (2022) [31]. SPROUT was a phase 3 multicenter, randomized, double-blinded, placebo-controlled study of 245 pediatric patients aged 6 to 17 with moderate-to-severe plaque psoriasis. At week 16, the endpoints were met: sPGA (33.1% vs. 11.5% placebo, *p* < 0.0001), PASI-50 (70.5 vs. 32.1% placebo), and BSA (56.6% vs. 21.8%, *p* < 0.0001). The safety profile of apremilast in this pediatric population remained consistent with previous studies, and no new safety concerns were identified.

### 4.2. Roflumilast

Roflumilast is a PDE4 inhibitor, similar in size to apremilast, with a chemical structure detailed in Table 2. Orally, it is known as Daliresp^®^ or Daxas^®^ and is utilized for the treatment of chronic obstructive pulmonary disease (FDA approval in 2011) [59]. Topical roflumilast (ARC-151; Zoryve^TM^) received FDA approval in 2022 and more recently in October of 2023 approved its use in children ≥ 6 years of age [60,61]. Health Canada approved topical roflumilast in 2023 for the management of plaque psoriasis including intertriginous psoriasis in adolescents aged ≥12 years and adults [62]. The topical formulation of roflumilast is delivered as a highly moisturizing, water-based cream, containing the cosmetic solvent diethylene glycol monoethyl ether or Transcutol^®^ [63]. It has a higher potency compared to other PDE4 inhibitors (25–300× more than apremilast or crisaborole depending on the comparator and PDE4 isoform [64]). The landscape of our knowledge on roflumilast is expanding, as evidenced by the growing body of clinical trials with various dermatologic concerns; notably, atopic dermatitis, seborrheic dermatitis, and psoriasis [25]. The clinical trial data for psoriasis are summarized in Table 2.

Among the earlier studies is a phase ½a single-dose open-label cohort and 28-day, double-blinded, randomized, cohort trial (NCT NCT03392168) reported by Papp et al. (2020) [51]. In the first cohort, eight patients applied a 0.5% roflumilast cream in an open-label setting. In the second cohort, 89 patients participated in a double-blinded study, randomly receiving either 0.5% or 0.15% roflumilast cream or a vehicle for 28 days. Patients had a history of >6-month chronic plaque psoriasis with ≤5% BSA. Both concentrations of roflumilast cream demonstrated significant improvements in the primary efficacy endpoint, the Target Plaque Severity Score (TPSS) × Target Plaque Area (TPA), achieving 66%–67% improvement compared to 38% with the vehicle at week 4. Adverse events, primarily mild-to-moderate (application site erythema/pain, nasopharyngitis, muscle strain), showed similar occurrence rates between the active treatment and vehicle groups, with no discontinuations due to side effects [51].

A subsequent investigation, phase 2b double-blinded, randomized trial (NCT03638258), was reported by Lebwohl et al. (2020) for the treatment of plaque psoriasis [50]. In this trial, 331 patients were randomized 1:1:1 (roflumilast cream 0.3%, 0.15%, vehicle) and the primary endpoint at 6 weeks was investigator global assessment (IGA) clear (0) or almost clear (1). Both 0.3% and 0.15% roflumilast creams proved significantly more effective than the vehicle cream: by week 6, 28% of those in the 0.3% group and 23% in the 0.15% group achieved clear or almost clear skin, compared to only 8% in the vehicle group (*p* < 0.001). Among the 15% of participants with baseline intertriginous psoriasis, 73% using roflumilast 0.3% cream, 44% with 0.15% roflumilast cream, and 29% with the vehicle achieved clear or almost clear skin, along with a 2-grade intertriginous area IGA score improvement. Baseline PASI scores were 7.7 for the 0.3% roflumilast group, 8.0 for the 0.15% roflumilast group, and 7.6 for the vehicle group, with changes at week 6 of −50.0%, −49.0%, and −17.8%, respectively. Pruritus and pruritus-related sleep loss were also improved with roflumilast (>4-point improvement in worse itch NRS compared to vehicle, *p* < 0.05, between weeks 2 and 12). Application-site reactions were low and exhibited similar patterns across all groups and adverse events included nasopharyngitis and upper respiratory tract infection. There was less than 1% incidence of nausea and diarrhea, less than what has been reported with apremilast [50].

The pivotal DERMIS-1 (NCT04211363) and DERMIS-2 studies (NCT04211389), as reported by Lebwohl et al. (2022) [47], constituted two identical phase 3 randomized, double-blinded, controlled, multicenter clinical trials involving 881 participants aged two years and older with plaque psoriasis covering 2–20% BSA. These studies investigated the daily application of roflumilast cream at a 0.3% concentration over 8 weeks. Statistically significant differences were observed in favour of roflumilast for various outcomes, including IGA (DERMIS 1—71.2% roflumilast vs. 13.8% vehicle; DERMIS 2—61.8% roflumilast vs. 18.5% vehicle) and PASI-75 (DERMIS-1—41.6% roflumilast vs. 7.6% vehicle [*p* < 0.001]; DERMIS-2—39.0 % roflumilast vs. 5.3% vehicle [*p* < 0.001]). Pruritus was also improved ≥4 points in WI-NRS as early as week 2 (DERMIS-1—34.9% roflumilast vs. 22.0% vehicle [*p* = 0.12]; DERMIS-2—41.2% roflumilast vs. 21.1 vehicle [*p* < 0.003]) and more so at week 8 (DERMIS-1—67.5% roflumilast vs. 26.8% vehicle [*p* = 0.001]; DERMIS-2—69.4% roflumilast vs. 26.8% vehicle [*p* < 0.001]). Adverse events, similar in the roflumilast and vehicle groups, were largely manageable, including transient gastrointestinal symptoms, headache, hypertension, and nasopharyngitis, with limited severe adverse events reported [47].

Subsequently, Papp et al. (2022) reported interim results from a 24-week open-label extension study (DERMIS-OLE; NCT04286607). There were two cohorts (*n* = 264), cohort 1 had completed a prior roflumilast study, and cohort 2 was naïve. In total, 84.1% of patients in cohort 1 completed the study, with 50.0% achieving IGA status of clear/almost clear at week 24, 43.8% achieving PASI-75, and 62.4% having 4-point improvement in WI-NRS. Safety and tolerability were consistent with previous studies, with only 0.4% discontinuing due to an adverse event, and 26.1% experiencing mild-to-moderate treatment-emergent adverse events.

Similarly, Lebwohl et al. (2023) presented at the Winter Clinical Dermatology Conference 2023 the results of the 52-week open-label extension of a phase 2b study (NCT03764475) [49,65]. Two cohorts were studied: cohort 1 (n = 230) completing 64 weeks of therapy and cohort 2 (*n* = 102) of naïve patients with at least mild psoriasis for over 6 months. After 52 weeks, the completion rate was 73.5%, with low discontinuations due to treatment emergent adverse events or inefficacy; most were due to withdrawal or loss of follow-up. The proportion of patients achieving IGA success was consistent: 34.8% for cohort 1, 39.5% for cohort 2. Intertriginous-IGA success was 60% at week 12, maintained by 66.7% of cohort 2 by week 52 (not recorded for cohort 1). Among those achieving clear skin, the mean response duration was 10 months. Topical roflumilast was well tolerated; most treatment emergent adverse events were mild or moderate, with 97% unrelated to treatment, and ≥97% showing no irritation on physician assessment.

Gyldenløve et al. (2023) recently reported the results of the PSORRO trial, a phase 2 multicenter, randomized, double-blinded, placebo-controlled trial (NCT04549870) involving 46 patients [48]. This study expanded the scope of roflumilast’s exploration in psoriasis by administering roflumilast orally at a dose of 500 µg once daily. Notably, the primary endpoint of PASI-75 was achieved, with 35% of participants demonstrating this level of improvement compared to 0% in the placebo group at week 12. This safety and efficacy profile persisted over 24 weeks. Common side effects, including transient gastrointestinal symptoms, weight loss, headache, and insomnia, led to the discontinuation of therapy in three patients [48].

At the 32nd European Academy of Dermatology and Venereology Congress in October 2023, the Phase 3 ARRECTOR trial (NCT05028582) presented its results [46]. This trial, designed as a randomized, double-blinded, vehicle-controlled study, enrolled 432 patients aged 12 years and above, investigating the use of roflumilast foam 0.3% for the treatment of scalp and body psoriasis in adults and adolescents. By week 8, roflumilast foam demonstrated significant improvements in scalp and body itch, with 65.3% of patients experiencing scalp itch relief with the achievement of ≥4-point itch NRS improvement from baseline in patients with baseline scalp itch NRS ≥4 compared to 30.3% in the vehicle group (*p* < 0.0001). Moreover, at week 8, 66.4% of individuals treated with roflumilast foam achieved S-IGA Success compared to 27.8% of those treated with the vehicle foam (*p* < 0.0001). Similarly, 45.5% of individuals treated with roflumilast 0.3% foam achieved B-IGA Success compared to 20.1% of those treated with the matching vehicle foam at week 8 (*p* < 0.0001). Adverse events, including headache, diarrhea, COVID-19, and nausea, were consistent with previous studies and led to few discontinuations [46]

### 4.3. Crisaborole

Crisaborole (AN2728; Eucrisa^®^) stands as a low molecular weight topical PDE4 inhibitor distinguished by a unique boron configuration within its molecular structure, which facilitates effective skin penetration [55,66]. (Table 2 and Table 3). Notably, it gained FDA approval for treating mild-to-moderate atopic dermatitis in 2016, followed by Health Canada’s approval in 2018, extending its use to individuals aged 3 months and older in 2020 [67,68]. While reports attest to its effectiveness in atopic dermatitis [39,57], its application in psoriasis, particularly when compared to apremilast and roflumilast, is limited (Table 1 and Table 2) [25]. It was investigated for psoriasis earlier on in its development. This overview provides a concise review of the existing literature on the use of crisaborole in the context of psoriasis.

One early report was presented at the 68th American Academy of Dermatology Annual Meeting in 2010 [52]. This double-blinded, vehicle-controlled clinical trial (NCT00759161) involved 35 subjects. Crisaborole (then AN2728) ointment 5% was applied to one psoriasis plaque, while an ointment vehicle was applied to a comparable plaque for 28 days. The results indicated that crisaborole 5% was well-tolerated and more effective (68.6% of crisaborole-receiving patients had lower scores compared to 5.7% of vehicle-receiving patients, *p* < 0.001) in reducing overall target plaque severity scores, erythema, scale, and plaque elevation in comparison to the ointment vehicle. These findings remained consistent at the 14-day, 21-day, and 28-day assessments, and no adverse events or laboratory abnormalities were noted.

For unpublished studies, early clinical trials included a phase 1 randomized, observer-blinded, single-center vehicle and comparator-controlled study (NCT00762658, completed in 2007) (Table 2) [25]. This study aimed to evaluate the dose-response relationship, efficacy, and safety of different concentrations of crisaborole ointment in 12 male subjects with stable psoriatic plaques. Each subject underwent examination of six test fields, encompassing three distinct concentrations of crisaborole ointment, a vehicle, a corticosteroid preparation, and a topical immunomodulator, with treatments administered over a 12-day period. Another phase 1 randomized, observer-blinded, single-center, vehicle and comparator-controlled initial dose-ranging study (NCT00763204, completed in 2008) involved 12 male subjects with stable psoriatic plaques, testing four different concentrations of crisaborole, an active ingredient-free vehicle, and a marketed corticosteroid preparation in an observer-blinded, randomized manner (Table 2) [25]. The test fields were treated over 12 days, with a total of 10 treatments administered, aiming to assess the antipsoriatic efficacy of AN2728 cream. Further, a phase 1 randomized, double-blinded, vehicle-controlled, multiple cohort study (NCT01258088, completed in 2010) enrolled white males aged 18 to 55, particularly individuals with psoriasis, although healthy volunteers were also accepted for the study (Table 2). This study sought to determine the extent of drug absorption throughout the body after topical application.

Unpublished phase 2 clinical trials were also completed. In a phase 2 single-center, randomized, double-blind, vehicle-controlled, bilateral design study (NCT00755196, finished in 2008), the objective was to assess the safety and efficacy of crisaborole 5% in comparison to an ointment vehicle when applied twice daily for 12 weeks in the treatment of plaque-type psoriasis (Table 2) [25]. Treatments were directed specifically to designated plaques, with all efficacy assessments focusing on these specific areas. Another phase 2 double-blinded, randomized, bilateral design study (NCT01029405, completed in 2010) aimed to assess the safety and efficacy of crisaborole, either 2% or 0.5%, compared to a placebo when applied once or twice daily for 12 weeks in patients with plaque-type psoriasis (Table 2) [25]. Eligibility criteria for the 145 enrolled participants included a clinical diagnosis of stable plaque-type psoriasis, among other factors. Finally, another phase 2 multicenter, randomized, double-blinded, 12-week clinical trial (NCT01300052, completed in 2011) aimed to assess the safety and efficacy of crisaborole in the treatment of mild-to-moderate plaque-type psoriasis [25]. The study included 68 participants, with the primary outcome measures focused on evaluating the improvement in psoriasis symptoms and the safety of the treatment over a 12-week period.

A recent article by Hashim et al. (2020) reported a double-blinded, randomized, vehicle-controlled trial with 21 participants to evaluate the effectiveness and safety of crisaborole 2% ointment in intertriginous, anogenital, and facial areas [69]. After 4 weeks of treatment, crisaborole showed 66% improvement in the Target Lesion Severity Scale (TLSS) compared to only 9% with vehicle (*p* = 0.0011). This improvement continued during an open-label phase, reaching 81% lesion improvement by week 8, with 71% of participants achieving clinical clearance (TLSS < 1). No adverse events were reported in this small sample size leading the authors to hypothesize that crisaborole may result in lower skin irritation rates in psoriasis [69].

Multiple case reports in the literature support the use of crisaborole for psoriasis in various locations, including the face, breasts, genitals, extensor and intertriginous areas, and palmoplantar regions [70,71,72,73]. For example, Lee et al. (2019) reported two cases: one involving a 58-year-old woman with persistent forehead psoriatic plaques that improved with daily crisaborole application, and the other featuring a 67-year-old woman with severe generalized psoriasis, notably on her face, groin, and under the breasts, showing significant improvement after 12 days of crisaborole use [70]. Similarly, Robbins et al. (2018) reported a case of a woman in her 50s with treatment-resistant palmoplantar psoriasis who achieved success with crisaborole [71]. More recently, Liu et al. (2022) detailed cases of men aged 26–45 with genital psoriasis [73] and a 29-year-old man with facial psoriatic lesions refractory to adalimumab [72], all treated effectively with crisaborole without complications.

### 4.4. Other PDE4 Inhibitors Investigated

Numerous other PDE4 inhibitors have been assessed in clinical trials for psoriasis, although there is relatively limited data published compared to apremilast, roflumilast, and crisaborole [25]. These PDE4 inhibitors include both oral and topical agents, including oral orismilast (LEO-32731), mufemilast (Hemay005), and ME3183 (Table 1), as well as topical PF-07038124, Leo-29102, MK-0873, and DRM02 (Table 2).

Orismilast, an oral PDE4 inhibitor, has been explored not only for psoriasis (Table 1) but also for atopic dermatitis and hidradenitis suppurativa during phase 1 and 2 clinical trials [25]. A two-part study reported recently by Warren et al. (2023) involved a prospective, randomized, double-blinded, placebo-controlled trial (NCT02888236) [43]. In the phase 2a segment, 36 patients with moderate-to-severe psoriasis were randomized to receive 30 mg of oral orismilast immediate-release (IR) or a placebo over a 16-week period. Results indicated that orismilast IR significantly improved the PASI score at week 16 compared to the placebo (7.1 vs. 13.1, *p* = 0.005). However, the orismilast IR group experienced a higher incidence of gastrointestinal-related adverse events than the placebo group (88.9% vs. 27.8%), resulting in a 50% discontinuation rate. In the phase 1 trial, orismilast-modified release (MR) was compared to orismilast IR in terms of pharmacokinetics. Orismilast MR exhibited similar pharmacokinetic properties, with a lower occurrence of gastrointestinal-related adverse events (16.7% vs. 33.3%) and no incidents of nausea. Consequently, the MR formulation was selected for further clinical development. The other clinical trials (NCT05190419; NCT03231124; NCT02126371) involving orismilast for psoriasis are unpublished [25].

Mufemilast, another oral PDE4 inhibitor, has been investigated for psoriasis (Table 1) [25]. Recently, Jones et al. (2023) presented the results of a phase 3 double-blinded, placebo-controlled clinical trial in China (NCT04102241) at the American College of Rheumatology Conference 2023 [44]. This trial enrolled 216 patients with moderate-to-severe chronic plaque psoriasis, finding the optimal dose of mufemilast was 60 mg PO BID and improvements in PASI-75 (53.6% with mufemilast vs. 16.0% placebo, *p* < 0.0001) and achieving sPGA success (31.3% mufemilast vs. 6.4% placebo, *p* < 0.0001) at 16 weeks. Most adverse events were mild and primarily gastrointestinal in nature. The results of an earlier phase 1 clinical trial examining the use of mufemilast for the treatment of psoriasis have not yet been published (NCT03007810).

The newest oral PDE4 inhibitor is ME3183, which had its first clinical trial completed in 2023 (NCT05268016) (Table 1) [25]. Recently, Papp et al. (2023) presented at the 32nd European Academy of Dermatology and Venereology the results of this phase 2a multicenter, randomized, double-blinded, placebo-controlled, parallel-group trial [74]. The study aimed to assess the efficacy and safety of ME3183 at various dosages (5 mg BID, 10 mg OD, 7.5 mg BID, 15 mg OD) for adult participants with moderate-to-severe plaque psoriasis. Primary endpoints were achieved with a significantly greater proportion of patients treated with ME3183 achieving PASI-75 at week 16 compared to a placebo (for non-responder imputation: 52.0% of patients treated with 15 mg OD, 61.5% 7.5 mg BID, 32.0% 10 mg OD, 58.3% 5 mg BID, 14.8% placebo; for last observation carried forward: 60.0% of patients treated with 15 mg OD, 65.4% 7.5 mg BID, 40.0% 10 mg OD, 58.3% 5 mg BID, 18.5% placebo). Early improvement was seen with approximately 50% reduction at week 4 and a greater proportion of patients who received ME3183 achieved PASI-90, PASI-100, and ssPGA 0/1. There were no unexpected safety concerns with adverse events including gastrointestinal symptoms, infection, hypertension, lipase increase, myalgia, and headache [74].

In terms of topical PDE4 inhibitors, PF-07038124 is a new compound that was designed using the previously studied and successful crisaborole as a model. It incorporates boron in its structure, hypothesized to enhance combinability with antibody therapies [75]. PF-07038124 has been investigated for atopic dermatitis and plaque psoriasis (Table 2): two phase 2 trials have been completed but results have not yet been published (NCT05375955; NCT04664153) [25]. Similarly, topical Leo-29102 has been previously investigated in phase 1 and 2 trials for atopic dermatitis and psoriasis (NCT00875277; NCT01466478) but there are no published reports [25]. Topical MK-0873 has undergone phase 1 trials for psoriasis (NCT01140061; NCT01235728) and phase 2 trials for rheumatoid arthritis, but there are no published reports [25]. Topical DRM02 has also been evaluated in phase 2 trials for rosacea, atopic dermatitis, and psoriasis (NCT01993433), with no published reports [25].

## 5. Discussion

Psoriasis, a chronic immune-mediated skin disorder, arises from a complex interplay of genetics, environment, and pathophysiology, substantially effecting the patient’s quality of life, with pruritus [3] and associated comorbidities [7] posing significant challenges. Treatment options encompass a spectrum from topical applications to oral and biologic systemic therapies, tailored to the severity of the condition. Given the global prevalence [4] and the substantial burden of psoriasis [5,6], innovative treatment approaches are important [10]. We reviewed the pathophysiology of psoriasis and the mechanism by which PDE4 inhibitors target cAMP to reduce the inflammatory response in psoriasis. This review also provided a comprehensive and up-to-date overview of the efficacy and safety of PDE4 inhibitors as a treatment for psoriasis with a specific focus on randomized clinical trials. We focused on the PDE4 inhibitors with the strongest evidence, including oral apremilast, topical roflumilast, and topical crisaborole.

Apremilast, FDA-approved for moderate-to-severe psoriasis, has demonstrated its efficacy and safety in the treatment of moderate-to-severe plaque psoriasis through a series of well-established clinical trials [27,28,29,30,32,33,35,36,37,38,41]. From early-phase studies [40,41] to phase 3 trials (ESTEEM 1 [38] and ESTEEM 2 [37]) and subsequent investigations [27,28,29,30,31,32,33,35,36,58], it delivered improvement, including PGA, PASI, quality-of-life improvements, and sustained benefits. Apremilast’s versatility extends to the treatment of various psoriatic manifestations, such as palmoplantar, nail, scalp, and genital psoriasis [32,58]. Most adverse events including diarrhea, nausea, and headache were transient, classified as mild-to-moderate in severity and resulted in low treatment discontinuation. Its favourable safety profile, including its use in pediatric patients [32], underscores its importance as a valuable therapeutic option. This substantial body of evidence establishes apremilast as a noteworthy addition to the treatment options for moderate-to-severe plaque psoriasis, with ongoing research and clinical trials planned for psoriasis (NCT04175613, NCT05863273, NCT06088199, NCT05565560, NCT02775500, NCT05601492, NCT06088043) and many other dermatologic conditions [25].

Roflumilast, in comparison to other PDE4 inhibitors, has demonstrated remarkable potency and effectiveness with topical application, leading to substantial improvements in psoriasis severity scores with reduced adverse events. Initial phase 1 and 2 trials [50,51] confirmed the efficacy and tolerability of topical roflumilast. The pivotal DERMIS-1 and DERMIS-2 phase 3 trials [47] and extension study [76] continued to validate its effectiveness, with minimal adverse events [47]. The ease of use with once daily dosing and applicability to all involved areas (body and intertriginous areas) provides a convenient topical solution for patients likely due to the differences in barrier dysfunction in eczematous and psoriatic skin. More recent reports have explored the application of oral roflumilast for psoriasis with significant improvements, albeit with some gastrointestinal side effects which may limit its use [48]. Roflumilast 0.3% foam was also investigated for scalp and body psoriasis, exhibiting substantial relief and success rates compared to vehicle foam [46]. Overall, these findings highlight roflumilast’s potential as a therapeutic option for chronic plaque psoriasis, whether applied topically or administered orally in more severe cases, with a predictable side effect profile. There is also a clinical trial for another topical roflumilast preparation for psoriasis that has been completed recently but not yet published (NCT05763082).

Crisaborole effectiveness in atopic dermatitis is well-established [39,57,67], but its application in psoriasis, particularly in comparison to apremilast and roflumilast, is more limited and it is unlikely that further research is planned. Early studies demonstrated the efficacy and tolerability of crisaborole [52]. Unpublished phase 1 and phase 2 clinical trials investigated dose-response relationships, antipsoriatic efficacy, safety, and drug absorption. Recent research by Hashim et al. in 2020 demonstrated the use of crisaborole in intertriginous, anogenital, and facial areas, suggesting its potential to reduce skin irritation in psoriasis [69]. Numerous case reports further support crisaborole’s versatility in treating psoriasis manifestations in various body areas including the face, breast, genitals, extensors, and intertriginous areas [24,70,71,73]. The thickness of the stratum corneum inversely affects the absorption of topical agents, making topical crisaborole well-suited for thin, non-scaly plaques [77]. While local burning and stinging sensations have been reported in atopic dermatitis, the pilot study in plaque psoriasis patients did not observe any adverse skin reactions, likely due to the inherent barrier dysfunction found in eczematous skin, which differs from psoriatic skin [69,78].

Several PDE4 inhibitors have undergone clinical evaluation for psoriasis, with limited data available for most in comparison to apremilast, roflumilast, and crisaborole (Table 1 and Table 2) [25]. Orismilast, a relatively newer oral PDE4 inhibitor, showed promising results in a recent phase 2a trial, improving PASI scores despite gastrointestinal adverse events and showing the MR formulation had improved tolerability [43]. Other trials are unpublished and there are no currently active or recruiting trials in progress [25]. Mufemilast, another oral PDE4 inhibitor, demonstrated efficacy in a phase 3 trial for psoriasis, leading to significant improvements in PASI and sPGA [44]. The other clinical trial results have not yet been published [25] but current active or recruiting clinical trials are expanding to investigate ankylosing spondylitis, atopic dermatitis, ulcerative colitis, and further investigation of psoriasis (NCT04839328) [25]. ME3183, the newest oral PDE4 inhibitor, recently completed its initial trial without published results. There are otherwise no other active or recruiting clinical trials currently [25]. Among topical PDE4 inhibitors, PF-07038124, Leo-29102, MK-0873, and DRM02 have been explored in trials for various skin conditions, but no published results in the context of psoriasis. For PF-07038124, there are additional clinical trials planned for PF-07038124 for vitiligo (NCT05298033), seborrheic dermatitis, and papulopustular rosacea (NCT06013371) but there are currently no trials recruiting for psoriasis [25]. Otherwise, Leo-29102, MK-0873, and DRM02 have no active or recruiting studies listed [25].

## 6. Limitations

This review, despite providing valuable insights into the utilization of PDE4 inhibitors for treating psoriasis, is constrained by limitations. The body of literature predominantly focuses on apremilast, roflumilast, and crisaborole as separate investigational entities. Recent randomized control trials have compared apremilast with risankizumab [26] and deucravacitinib [79] for psoriasis treatment. There are otherwise few direct comparative studies among the PDE4 inhibitors with other topical or systemic treatments thereby limiting our ability to assess their relative efficacy and safety comprehensively.

There is also a paucity of published results for some of the clinical trials, notably those involving crisaborole and other PDE4 inhibitors such as orismilast, mufemilast, ME3183, PF-07038124, Leo-29102, MK-0873, and DRM02. Another noteworthy limitation pertains to extended-term data; many of the studies are characterized by relatively short durations and limited participant numbers. Furthermore, there is population heterogeneity, including differences between female and male patients and distinctions between pediatric and adult populations. However, some patient groups, such as pregnant individuals, remain underrepresented. Additionally, many investigations involve plaque psoriasis, with limited insights available regarding non-plaque variants.

## 7. Conclusions

The burden of psoriasis necessitates the exploration of broad therapeutic options, with a particular focus on cAMP targeted by PDE4 inhibitors to reduce the inflammatory cytokine secretion associated with psoriasis as an expanding avenue for treatment. Clinical trials in moderate-to-severe psoriasis show promise, notably in the case of oral apremilast and topical roflumilast, which are both approved for plaque psoriasis. Apremilast consistently demonstrated efficacy, safety, and versatility in addressing diverse psoriatic manifestations with some mild-to-moderate adverse events. Whether administered topically or orally, roflumilast offers an effective treatment option with manageable side effects that were seen with higher frequency when administered orally. While crisaborole is approved for use in atopic dermatitis, its potential extends to various psoriatic presentations. These PDE4 inhibitors lay the foundation for an optimistic future in psoriasis management. The ongoing expansion of clinical trials, as well as continued research on existing agents and the development of novel inhibitors, holds the potential to broaden therapeutic choices for psoriasis and enhance outcomes.

## Figures and Tables

**Table 1:** Oral phosphodiesterase-4 inhibition clinical trials completed for psoriasis. Information retrieved from [25].

PDE4 Inhibitor	Formulation	Sponsor	Condition	Phases	NCT Number (Year Completed; Bolded Randomized Control Trials)
**Apremilast (CC-10004)**	Oral	Amgen (Boca Raton, FL, USA)	Psoriasis; Plaque psoriasis; Nail psoriasis; Palmo-plantar psoriasis	IV	NCT06032858 (2023); **NCT04908475 (2023) [26] NCT03774875 (2021) [27];** NCT03082729 (2021); NCT03022617 (2020); NCT03000309 (2018); NCT03441789 (2018); NCT02412644 (2016); **NCT02400749 (2016) [28]; NCT02425826 (2016) [29,30]**
				III	**NCT03701763 (2023)** [31]; NCT06084663 (2022); **NCT03777436 (2022) [32];** NCT03930186 (2020); **NCT03721172 (2020) [33];** NCT03930186 (2020) [34]; **NCT03123471 (2019) [35]; NCT01690299 (2016) [36]; NCT01232283 (2016) [37]; NCT01194219 (2016) [38]**
				II	NCT03442088 (2021); NCT02576678 (2019); NCT02576678 (2017) [39]; NCT01988103 (2014); **NCT00773734 (2009) [40];** NCT00521339 (2009); **NCT00606450 (2007) [41];** NCT00604682 (2005) [42]
**Orismilast (LEO-32731)**	Oral	LEO Pharma; Union therapeutics (Hellerup, Denmark)	Psoriasis; Psoriasis vulgaris	II	**NCT05190419 (2022); NCT02888236 (2017)** [43]
				I	**NCT03231124 (2017);** NCT02126371 (2015) **NCT02514694 (2015); NCT02888236 (2017) [43]**
**Mufemilast (Hemay005)**	Oral	Tianjin Hemay Pharmaceutical Co., Ltd. (Tianjin, China)	Psoriasis	II	**NCT04102241 (2021) [44]**
				I	NCT03007810 (2018)
**ME3183**	Oral	Meiji Pharma USA Inc. (Santa Ana, CA, USA)	Plaque psoriasis	II	**NCT05268016 (2023)**

NCT—National Clinical Trial.

**Table 3:** Summary of phosphodiesterase-4 (PDE4) inhibitor properties and adverse events: apremilast, roflumilast, and crisaborole.

PDE4 Inhibitor	Apremilast [53] (CC-10004) Otezla^®^	Roflumilast [54] (ARQ-151) Zoryve^®^	Crisaborole [55,56] (AN2728) Eucrisa^®^
Molecular formula and 2D structure	C_22_H_24_N_2_O_7_S 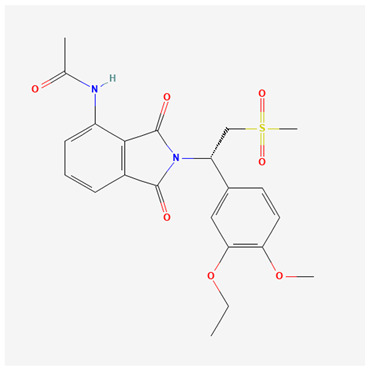	C_17_H_14_Cl_2_F_2_N_2_O_3_ 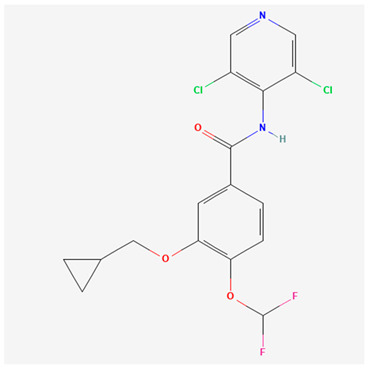	C_14_H_10_BNO_3_ 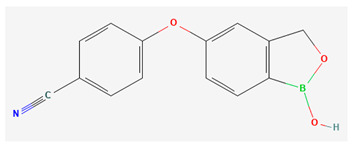
Molecular Weight	460.5 g/mol	403.2 g/mol	251.05 g/mol
logP	1.8	4.6	2.6
Formulation, dose	Oral: 30 mg twice daily	Topical: 0.3% once daily to affected areas Oral: 500 mcg once daily	Topical: 0.5% or 2% once daily to affected areas
Adverse events	Nausea, vomiting, diarrhea, headache, nasopharyngitis, dyspepsia, upper respiratory tract infection, loss of appetite, weight loss [27,28,29,30,32,33,35,36,37,38,41]	Application site erythema/pain, nasopharyngitis, upper respiratory tract infection, muscle strain, gastrointestinal symptoms, weight loss, headache, insomnia [46,47,48,50,51]	Mild application site reaction [57]

## Data Availability

No new data were created or analyzed in this study. Data sharing is not applicable to this article.
